# Concluding Notes—Looking Back; Looking Forward

**DOI:** 10.1007/s10936-025-10156-2

**Published:** 2025-05-26

**Authors:** Wilma Bucci

**Affiliations:** Derner Institute, Adelphi University, 1 South Ave, Garden City, NY 11530 USA

**Keywords:** Subjective experience, Mathematical foundations, Function words, Smoothing process, Weighted computerized dictionaries, Clinical research

## Abstract

This summary paper provides a brief review of major contributions in a group of papers that were collected to honor the work of Bernard Maskit, who died in March 2024. Professor Maskit was a mathematician who contributed major theoretical and methodological advances in the study of multiple code theory and the referential process. Each of the papers in this issue incorporate applications of his ideas and the new measures that he developed. The applications include basic epistemological ideas, linguistic findings, and innovative approaches to psychotherapy theory and research. Plans for future theoretical and applied research directions are discussed.

## Introduction

The papers included in this issue have focused on the process of connecting experience and language, and on the intersection of theory and measurement in examining this process. The multiple code theory on which this work is based has its roots in a broad range of fields, including cognitive science, neuroscience and linguistics, as well as psychotherapy; the work presented here has the potential to contribute to findings in all these areas. The process we are studying is one that researchers can observe within themselves, drawing hypotheses from their own subjective experience; that is both a strength and a weakness of our approach. In their paper in this issue, Negri and Barazzetti have viewed this feature and its implications from an epistemological perspective.

The weighted measures that are applied in our research were based on the subjective experience of raters judging qualities of texts. From an alternate perspective, the measures also depend on the implementation of complex mathematical processes that are accessible only to a subset of trained mathematicians. The intersection and mutual facilitation of the psychological and mathematical processes underlying our work were emphasized in the paper by Maskit that was the central presentation of the conference on which this special issue is based. The mathematical foundations of these processes and their implications were also discussed from a somewhat different perspective in a paper by Susskind.

As noted in the introduction to the issue, there has been only a small and disparate field of research on the central question with which our work is concerned—how people generate language in the moment of speaking or writing. In addressing this question, we referred briefly to the perspectives of several developmental psychologists, including Piaget (1950), Bruner (1966) and Vygotsky (1986), who recognized different aspects of the question and produced a range of different formulations.

In the context of his work on thought and language, the Russian neuropsychologist Luria (1966) offered a detailed outline of the process of connecting these functions. He described a sequence of phases, beginning with *inner language*, moving from there to *semantic set representations*; and *deep syntactic structures*, and then to the *serial encoding of surface language*. Emotional and bodily experience are however not included in the domain of inner language as formulated here.

From another perspective, psychoanalytic theory also focuses on the process of connecting experience and language; in this context the retrieval and verbal communication of thoughts or memories that are linked to dreaded experiences and fantasies. Rather than a distinction between inner language and surface forms, the psychoanalytic theory of mind is largely defined by the distinction between the contents and formats of conscious and unconscious representations and the difficulties involved in bringing unconscious experience to awareness.

In contrast to both of these perspectives, the multiple code theory incorporates a distinction between *nonsymbolic* and symbolic mental functions, which may be *conscious or unconscious*, and which continue throughout life, and are present at every stage of development. The difficulties people experience in generating spoken or written language arise from the problem of connecting the widely disparate forms of nonsymbolic experience, occurring simultaneously in multiple channels, with the single channel symbolic forms of the verbal code. The nonsymbolic experience may include emotional or bodily experience that is neutral or positive as well as painful experience that is dreaded and kept out of awareness.

As Leon Hoffman emphasizes in his paper, the multiple code theory, with its corollary concepts of the referential process and the emotion schemas, has provided a systematic formulation of the functions involved in linking painful emotional experience to words that is compatible with a psychoanalytic approach. The tools developed by Maskit and expanded by others in our group have enabled empirical study of these functions. Each of the papers in this issue has offered new insights concerning this process and new applications of it.

## Basic Linguistic Findings of the DAAP Procedures; Intended and Unintended

In their overview of language evolution, Tallerman and Gibson (2013) emphasized two major features that distinguish human language from the communication systems of other species: the ability to learn and use categories of function words, such as pronouns, determiners, conjunctions, and prepositions; and the capacity to combine words in different ways to produce infinite sets of meanings. Both of these features are central in the development of the referential process measures, as outlined by Maskit and Susskind in their papers.

*The power of function words. C*omputerized language dictionaries usually eliminate these function words; the widely used LIWC (Pennebaker et al., 2001) includes a general category of function words but does not distinguish among them. Maskit’s system of developing weighted dictionaries allowed identification of the use of these limited categories of words in the different modes of expression in the referential process, as presented in his summary paper.

The most powerful function words in the WRAD dictionary, the basic measure of the narrative symbolizing phase, are the words that people use when telling stories and describing images; using these words, experiences of the past emerge in image or story form. The most powerful function words in the WRRL, the weighted measure of reflecting on and reorganizing experience, are words that people use to relate to another person and to refer to themselves, in the present. In the WRSL, the measure of the Arousal function, speakers use first person pronouns to refer to themselves and also use the item ‘mm’, which indicates a filled pause.

*The effects of word order*. The effects of different ways of combining words also emerged indirectly, from Maskit’s introduction of the mathematical technique called ‘smoothing’. As the mathematician Susskind notes in his paper in this issue, this innovation ‘provides an almost deictic aspect in evaluating each word of a text’; i.e., through the smoothing process, the meaning of any word or any expression depends not on itself alone but on the order of the surrounding words. When a speaker or writer expresses an idea, they do not choose this word order any more than they choose the function words themselves.

Through the combination of the weighting and smoothing techniques, and several related processes, the development of the DAAP system has provided new evidence concerning both the power of function words and the effects of word order in distinguishing modes of expression of experience as characterized by the functions of the referential process. These features of language usage, which are not chosen by the speaker, emerged indirectly as byproducts of the DAAP procedures. As emphasized earlier, these features of human language have not been identified in any of the language forms that can be acquired by other species.

### The Rhythms of Speech; Contributions of the Time-DAAP

In her paper in this issue discussing the development of the Arousal measure and presenting early applications, Tocatly emphasized that pausing and disfluent speech were important indicators of the struggles involved in this function, going beyond the words themselves. The measurement of these features of spoken language have now been made possible by the new TimeDAAP as described by Maskit in this issue.^1^ Early work applying TimeDAAP to study these processes and their measurement is also presented in the paper by Peterson in this issue.

## Contributions to Clinical and Psychotherapy Theory and Research

As Susskind pointed out in his paper on mathematical aspects of the DAAP, the measures and their graphic representations provide insights concerning the flow of language from different perspectives—within the speaker’s experience and outside of it. The measures and graphs provide a representation of the speaker or writer’s experience in generating language; carried out largely without attention or intention. From the viewer’s perspective, the measures have the potential to provide the researcher or clinician with a systematic perspective of the underlying meaning and intent of the language that the speaker *does not have*.

Given these different perspectives, the measures provide a set of tools that can be used in many different ways with a number of different applications. Much of our work has been carried out in clinical settings –in studies of the relation of linguistic functions to emotional and neural disorders in clinical samples, and in psychotherapy research. Both of these types of applications are represented in this issue; these applications, and others in process or in planning, are needed to test and revise the theory.

Several papers have examined the basic concepts of multiple code theory from different clinical perspectives. Leon Hoffman has compared the multiple code concept of emotion schemas to the psychoanalytic concept of unconscious fantasies. From a contrasting empirical perspective, Mariani and her colleagues have emphasized the somatic components of the organization of emotional experience, and provided evidence for this organization using the Italian versions of the DAAP (the IDAAP). Their work points to dissociation among components of the emotion schemas as associated with a range of somatic disorders, including hypertension, infertility difficulties, and anorexia nervosa. As they note, somatic symptoms may operate to express underlying distress that cannot be spoken—or even recognized as emotional. While the physical symptoms may be real, the work of therapy involves attending to its emotional meaning as well. In an earlier paper, Solano (2010), one of the co-authors of that paper, has provided support for these findings from the perspectives of psychoanalytic theory as well as multiple code theory.

## Contrasting Approaches to Therapy Research

While the studies by Mariani and her colleagues examined the interaction between somatic disorders and linguistic expression in large and varied clinical samples, the paper by Christian et al. provides an example of what can be learned about such interaction in the intensive study of a single case, a patient diagnosed with Body Dysmorphic Disorder (DSM-5-TR). The physical nature of the patient’s presenting complaint was and remains unclear; the goals of treatment focused on aspects of self-organization and relation to others, as well as reduction of depressive symptoms. Measures of personality organization, mental functioning and symptom patterns showed changes from early to middle stages of the treatment; these changes were also represented in the language measures. The patterns of change in the course of this patient’s engagement in the treatment and the therapist’s contribution to this change can be seen directly in the graphs of the process comparing sessions from the two periods of the treatment.

In the complete brief treatment presented by Jaffe, we can observe the different phases in the course of the treatment from the perspective of the patient’s speech as represented in the measures, and also from the perspectives of the therapist’s reactions. Jaffe’s study identified turning points in the treatment as a whole and also looked within each session to see how the process played out. For this case, the therapist also provided his view of the patterns of change independent of the measures; the findings reveal correspondence between his views of the process and the results shown by the measures, in individual sessions, and in segments of the treatment.

In contrast to these papers, each of which focused on a single case, and incorporated the perspective of the treating analyst, the study by Hong and Watson was based on a sample of sessions taken from brief treatments, including 20 CBT and 20 EFT treatments as identified in earlier research, with each including 20 good and 20 poor outcomes, based on widely accepted change measures (for a total of 80 sessions). Contrary to their predictions, they did not find a difference in the patients’ symbolizing processes or in their capacity to reflect on their experience between the two outcome groups; their major findings concerned the language of the therapists rather than of the patients. In particular, therapists in the highest rated sessions showed higher levels of the WRAD measures of the symbolizing function, as well as greater fluency. The authors suggest that the findings concerning the patient language may have been limited by the selection of only two sessions per client in each treatment rather than covering the variation over the course of the treatments more fully.

The study by Tocatly, which focused primarily on psychoanalytic treatments, also produced findings concerning therapist language. She investigated interactions in sessions from six psychodynamic treatments drawn from the Referential Process Database, applying a program specifically developed by Bernie Maskit to identify sessions of interest for the study’s analysis. Among other results, she found that therapist interventions that were most effective in facilitating patient movement from searching to finding (from Arousal to Symbolizing) employed language high in Reflecting/Reorganizing (R/R) features—that is, language that was reflective, emotionally engaged, and centered on meaning-making.

## Going Forward: The Research of the Future

The studies included in this issue have applied the DAAP measures in several research designs with different categories of participants and treatment forms, and have provided some overlapping and some diverging results. Going forward, we expect the measures will provide the basis for a broad range of studies that include samples of complete treatments with systematic process and outcome assessment and that also incorporate the therapist’s perspective. Such studies can now readily be carried out by treatment researchers. The DAAP program and RP dictionaries can be downloaded from the referential process website (www.referentialprocess.org) and automatized transcribing procedures are widely available.

The use of our measures in treatment research that adequately represents the values of the psychodynamic approach and meets accepted research standards has been a goal of our project from the beginning. Such studies could also include forms of measurement-based care that provide regular feedback to therapists and patients. Routine monitoring of this nature has often been seen by psychodynamic clinicians as not compatible with the nature of their work, both practically and philosophically. In contrast to patient self-report or therapist assessments, the DAAP measures can assess the course of treatment without risk of encroaching on the process or the relationship. Regular assessment using these measures has the potential to provide useful information for practitioners as well as aligning psychodynamic treatment more closely with the world of evidence-based practice.

There is also increasing interest in treatment research in residential treatment centers that focus on the psychodynamic approach. The use of our measures in such institutions would have considerable value for them in monitoring treatment. The application in such institutions also has the potential to provide validation for the language measures based on the extensive psychological and physiological assessment that is routinely carried out in such institutions.

Using the new TimeDAAP in any of these settings will enable adding measures of pausing and speech rhythms, and also open access to methods that register changes in vocal register, including loudness and intonation patterns. These measures will provide a fuller picture of the therapeutic relationship, as well as the effectiveness of the treatment process.

### Looking to the Future

I am writing this conclusion as the originator of the multiple code theory that provides the theoretical framework for the work presented at the conference and in this issue. I was also the life-, as well as work-partner, of Bernard Maskit, who created the measures used in this research. He was continuously looking for the best way to represent the ideas of multiple code theory and the referential process in numerical and graphic form and to expand these ideas towards new applications. My own experiences in working with Bernie often included my expressing a wish for a new measure or new application that was needed in our research, Bernie saying ‘no that is not possible’, and then producing the measures.

In addition to the application of the DAAP measures in therapy process and outcome research, our goal from the beginning of our project has been to develop measures that can be directly relevant and useful for therapists in their ongoing clinical work. Along these lines, an early fantasy, which motivated aspects of our work, was that a clinician could go into their office, push a button, and at the conclusion of a session produce a transcript and a graph that represented the word-by-word (or moment-by-moment) process of the session, as well as numerical summary data. The clinician could then examine the graph to identify aspects of the process that appeared problematic, or particularly effective. They could also compare the progress across the treatment, as seen in the graphs and the summary measures. This process would be much the same as a cardiologist examining cardiograms or a neurologist examining brain scans—equally standard procedure in understanding a case and equally necessary for appropriate treatment.

A central aspect of this wish has now been fulfilled. A version of the DAAP program, the DAAPLab, developed by Attà Negri and his colleagues (also available on the referential process website) has been designed to provide a more user friendly interface that can also run multiple languages. The outputs of this new program have been compared to DAAP and are highly reliable. The program allows clinicians to produce summaries of measures and graphs of sessions for their own use in monitoring a treatment, as well as for research purposes. (The DAAPLab does not yet produce derived measures; those are in the works.)

The program has been applied by Christian for supervision purposes; he writes “It takes 20 seconds to run it and get the graph I use. And it’s all done by the click of a mouse. 9 clicks to be exact.” (personal communication). In working with a supervisee, the supervisor can look at the graph of a session, note a moment that seems significant but that the supervisee might not previously have noted in their report of a session and open a discussion of ‘what happened then?’.

Bernie continued developing new skills and ideas to the last days of his life. He left us, as part of his legacy, a number of ideas for other projects that he planned to do, including many works in progress for the TimeDAAP, as well as the construction of new weighted dictionaries, including Affect dictionaries, using techniques similar to those applied in developing the weighted measures of the referential process. Some of these ideas are included in his paper in this issue; other ideas and directions are presented in papers by our colleagues. This collection of papers has been made possible by colleagues carrying forward the work and by the generative nature of the collaborative process. We look forward to new applications, new results, and the emergence of new fantasies to be realized.

## Footnotes

1 Michael Peral worked closely with Bernie Maskit in development of the TimeDAAP, including implementation of the Google speech to text program that was basic for development of the features of the new program.
